# Comparative Transcriptome Analysis Reveals Stem Secondary Growth of Grafted *Rosa rugosa* ‘Rosea’ Scion and *R. multiflora* ‘Innermis’ Rootstock

**DOI:** 10.3390/genes11020228

**Published:** 2020-02-21

**Authors:** Jing-shuang Sun, Rui-yang Hu, Fu-ling Lv, Yan-fang Yang, Zhi-min Tang, Guang-shun Zheng, Jian-bo Li, Hua Tian, Yan Xu, Shao-feng Li

**Affiliations:** 1 Experimental Center of Forestry in North China, Chinese Academy of Forestry, Beijing 102300, China; sjshuang1129@163.com (J.-s.S.); hury1102@163.com (R.-y.H.); lflshiplmm@126.com (F.-l.L.); tangzm9603@sohu.com (Z.-m.T.); guangshunzheng@163.com (G.-s.Z.); lkyuan101@163.com (J.-b.L.); happyth1949@sina.com (H.T.); xuyan19932019@163.com (Y.X.); 2 Research Institute of Forestry, Chinese Academy of Forestry, Beijing 102300, China; echoyyf@caf.ac.cn

**Keywords:** RNA-sequencing, *Rosa*, grafting, stem, scion, rootstock

## Abstract

Grafted plant is a chimeric organism formed by the connection of scion and rootstock through stems, so stem growth and development become one of the important factors to affect grafted plant state. However, information regarding the molecular responses of stems secondary growth after grafting is limited. A grafted *Rosa* plant, with *R. rugosa* ‘Rosea’ as the scion (Rr_scion) grafted onto *R. multiflora* ‘Innermis’ as the stock (Rm_stock), has been shown to significantly improve stem thickness. To elucidate the molecular mechanisms of stem secondary growth in grafted plant, a genome-wide transcription analysis was performed using an RNA sequence (RNA-seq) method between the scion and rootstock. Comparing ungrafted *R. rugosa* ‘Rosea’ (Rr) and *R. multiflora* ‘Innermis’ (Rm) plants, there were much more differentially expressed genes (DEGs) identified in Rr_scion (6887) than Rm_stock (229). Functional annotations revealed that DEGs in Rr_scion are involved in two Kyoto Encyclopedia of Genes and Genomes (KEGG) pathways: the phenylpropanoid biosynthesis metabolism and plant hormone signal transduction, whereas DEGs in Rm_stock were associated with starch and sucrose metabolism pathway. Moreover, different kinds of signal transduction-related DEGs, e.g., receptor-like serine/threonine protein kinases (RLKs), transcription factor (TF), and transporters, were identified and could affect the stem secondary growth of both the scion and rootstock. This work provided new information regarding the underlying molecular mechanism between scion and rootstock after grafting.

## 1. Introduction

Grafting is an ancient plant asexual propagation technique. It is practiced in forest trees, fruit trees, vegetable crops, and in many ornamentals. There are many advantages of using grafted plants, such as yield increase, stress tolerance, and successive cropping, which have been well-studied for several decades [1,2]. The rootstock, i.e., the entire root system of a grafting plant, is often selected to enhance nutrient uptake and alter various physiological processes in the scion, such as biomass accumulation [3], fruit quality [4], and response to abiotic stresses (e.g., water deficit and salinity) [5,6]. Little published information regarding the effect of the scion on rootstock development after grafting is available, but the scion genotypes that confer rootstock vigor and root patterns have been identified [3]. Grafting techniques are increasing for many plants around the world.

The stem of the scion and rootstock have important roles in the growth and development of the whole grafted plant, especially the development of the secondary vascular tissue of the shoots [7]. In recent studies, changes in the vascular anatomy and the role of some hormones in the vascular differentiation of the stems between the scion and rootstock have been identified during the grafting process [8,9]. Rootstocks used in apple orchards reportedly have dramatic dwarfing effects on the scion, reducing the trunk diameter by up to 70% [10]. Cookson [11] verified that some selected rootstocks could alter the secondary growth (stem thickness) rather than the primary growth (stem length) in a grafted grapevine. However, the mechanisms underlying stem development changes in grafted plants are insufficiently understood.

Previous studies have reported that physiological differences are strongly correlated with gene expression changes in the scion after grafting. Transcriptional profiling of the scions of *Malus domestica* revealed that differences in the expression of 116 transcripts were correlated with rootstock regulation of tree size in the scion [12]. In citrus, most differentially expressed genes (DEGs) of ‘Shatangju’ mandarin (*Citrus reticulata* Blanco) leaves were involved in the auxin signal transduction pathway and gibberellin (GA) biosynthesis pathway in grafted plants [13]. The whole-leaf transcriptome of a grapevine grafted onto five selected rootstocks showed high variability in gene expression, and 2692 DEGs and metabolites were involved primarily in defense responses (Chitarra [14]. The tree size of the ‘Shatangju’ mandarin scion was found to be largest when grafted onto Canton lemon, and more than 1000 DEGs were found to be involved in the oxidoreductase function, hormonal signal transduction, and the glycolytic pathway in red tangerine vs. Canton lemon [13]. However, molecular regulation of the grafting effect on stem secondary growth has seldom been studied.

*Rosa* spp. are among the most popular ornamental plants worldwide. In previous studies of *Rosa* grafting, grafted roses have usually been found to have high productivity due to the effect of the stock on nitrogen metabolism [15]. The number of days from grafting to the basal axillary bud break of the scion in *R. hybrida* was shown to be affected by the genotype of the stock, most likely via its cytokinin production [16]. The winter tolerance of *Rosa* cultivars was improved when they were grafted onto *R. multiflora* [17]. One new application of grafting in *Rosa* is the creation of the tree rose, in which *R. hybrid* or *R. rugosa* cultivars are used as scions grafted onto a higher (1 to 1.5 m) *R. multiflora* ‘Innermis’ rootstock. In this study, we found that the stem thickness of the rootstock was significantly increased when grafted with *R. rugosa* ‘Rosea’ after 1 year, compared with ungrafted rootstock as a control. However, the underlying molecular mechanisms remain obscure. To elucidate the grafting effects on the thickness of rootstock and scion, we analyzed the physiological and morphological characteristics of the stems in Rr_scion and Rm_stock compared with ungrafted ones. To further investigate the molecular mechanism behind the increased stem thickness, RNA-Seq was applied to identify differentially expressed genes (DEGs). The transcriptome data presented here provide straight forward information regarding the molecular state of grafted plants, which is important for understanding the transcriptomic changes of grafted Rosa plants.

## 2. Materials and Methods

### 2.1. Plant Material and Growth Conditions

Seedlings of *R. multiflora* ‘Innermis’ and *R. rugosa* ‘Rosea’ were planted in a greenhouse at the Experimental Center of Forestry in North China, Chinese Academy of Forestry, at a temperature of 24 ± 1 °C and relative humidity of 50 ± 10%. When the buds of *R. rugosa* ‘Rosea’ were full and vigorous, their stems were cut, and a plant section consisting of the buds and a short length of trunk were grafted onto rootstocks of the same height. The grafted seedlings were covered with plastic film and monitored to maintain a daytime temperature of 25–30 °C and nighttime temperature of 15–20 °C, with humidity maintained at 60–80% for 30 days and then reduced to 30–50% thereafter. In July, the scions and rootstocks were both in a steady state, and the grafts had formed a successful union [11]. Different samples were designated Rm_stock (*R. multiflora* ‘Innermis’ as stock), Rr_scion (*R*. *rugosa* ‘Rosea’ as scion), Rm (*R. multiflora* ‘Innermis’ ungrafted), and Rr (*R*. *rugosa* ‘Rosea’ ungrafted). Stems were separately harvested from the seedlings, Stem samples of Rm_stock and Rm were collected at the same position as well as Rr_scion and Rr: Rm_stock (5 cm distance from the branch point, about 5–8 cm distance from the graft union), Rm (5 cm distance from the branch point), Rr_scion (between the tenth and twelfth leaf, about 5–8 cm above the graft union), Rr (between the tenth and twelfth leaf). Rr_scion, Rr, Rm_stock, and Rm as samples for transcriptome analysis. These were quickly frozen in liquid nitrogen and then stored at −80 °C until further use. All experiments included three to five grafted plants under each treatment, and all were repeated three times. The stem diameter (the sixth internode) was measured in Rr_scion and Rr; for Rm_stock and Rm, the stem (about 5 cm below the grafting point) and root neck were measured. In addition, the lignin and cellulose content of stem dry matter in Rm_stock, Rm, Rr, and Rr_scion were determined by visible spectrophotometry (Solarbio, Beijing, China). Between Rr_scion and Rr, Rm_stock and. Rm, statistical analysis to find significant differential content of both lignin and cellulose was determined using a two-tailed Student’s *t*-test in Microsoft Office Excel 2017 (*p*-values < 0.05 α-level).

### 2.2. Histological Analysis

The stems of Rr_scion, Rr, Rm_stock, and Rm were cut at the same measurement position. Tissue sections were prepared using a slicer (VT1200, Leica, Wetzlar, Germany) and then stained with 0.05% toluidine blue for 1 min to visualize secondary xylem tissues. The images were recorded using a Zeiss Axioplan light microscope (Zeiss, Jena, Germany), and images were captured using an axiocam digital camera (Zeiss) and AXIOVISION v.4.5 software (Carl Zeiss Vision GmbH, Hallbergmoos, Germany). The xylem width was measured from the border of the cambium flanking the xylem to the outermost cells of the pith [18].

### 2.3. Construction of the cDNA Library and Solexa Sequencing for Transcriptomic Analysis

For all samples, total RNA was extracted using TRIzol (Invitrogen, Carlsbad, CA, USA) as per the manufacturer’s protocol. The quality and concentration of each RNA sample was assayed using 1% agarose gel electrophoresis, a spectrophotometer (K5500, Kaeo, China), and a bioanalyzer (2100 RNA Nano 6000 assay kit, Agilent Technologies, Santa Clara, CA, USA). Poly(A) mRNA was isolated using magnetic oligo(dT) beads, and the isolates were then divided into short fragments (Ambion, Austin, TX, USA). The first-strand cDNA was synthesized using random hexamer primers, and the second-strand cDNA was synthesized using dNTPs, buffer, RNaseH (Invitrogen), and DNA polymerase I (New England Biolabs, Ipswich, MA, USA). Short fragments were purified, subjected to end repair and the addition of poly(A), and were then ligated to sequencing adapters. The fragments of interest were purified with agarose gel electrophoresis and enriched using polymerase chain reaction (PCR) amplification. Finally, all cDNA libraries were sequenced using an Illumina Hiseq4000 platform with a PE150 sequencing strategy.

### 2.4. Sequencing, Assembly, and Functional Annotation of cDNA

Raw data (Raw reads) was firstly processed by with Perl Scripts. In this step, clean data (reads) of fastq format were obtained by removing reads with any of the following: (1) adapter contamination > 5 bp; (2) both more than a 15% base calling accuracy, a Phred quality score Q ≤ 15; (3) reads with number of N base accounting for more than 5%; As for paired-end sequencing data, both reads will be filtered out if any read paired-end reads are adaptor-polluted. Transcriptome assembly was accomplished using Trinity method (20140717) [19]. The obtained clean data after filtering will be carried out on statistical analysis, Q30, GC-content and sequence duplication level of the clean data were calculated.

We predicted open reading frames (ORFs) of the assembled transcripts using TransDecoder (v20140717) based on the following criteria: a minimum length of 100 amino score and greater than 0 is reported; if a shorter ORF is fully encapsulated by a longer ORF, the longer one is reported; Trinotate (20140717) was used for performing the functional annotation of ORFs on the base of database. We predicted the unigene homology using the BLAST database (2.2.28), protein signal peptide and transmembrane domain prediction using SignalP (4.1) and TmHMM database (2.0), and then compared these outputs to current annotation databases, UniProt protein database, eggNOG database (4.5.1). The results were controlled by an e-value threshold of 10^−5^. Gene ontology (GO) annotations of contigs were determined using Blast2GO (http://www.blast2go.com/) according to molecular function, biological process, and cellular component ontologies (http://www.geneontology.org/) [20]. Here, we used an e-value threshold of 10^−5^. Annotated contigs were used to query the KEGG to define the KEGG orthologs (KOs). The KEGG mapping tool was used to plot these KOs into the complete metabolic atlas.

### 2.5. Identification of DEGs and Functional Analysis

The read counts for each gene in each sample was counted by high-throughput sequencing data (HTseq-count) (v.0.6.0). We applied the DESeq2 (V.1.4.5) method to analyze the expression of contigs between the test and control treatments of our experiment, and a model based on the negative binomial distribution was performed for normalization [21]. The *p*-value was assigned using Benjamini and Hochberg’s approach for controlling the false discovery rate [22], The DEGs were selected if their |log_2_Ratio| ≥ 1, and *q* < 0.05.

DEGs were functionally annotated using the UniProt, Pfam, GO, and KEGG databases. A GO enrichment analysis was performed using Blast2GO, and we detected which of the DEGs were significantly enriched in GO terms using *q* < 0.05 as the threshold of significance. The cellular metabolisms, biochemical pathways, and biological potential of DEGs in the KEGG pathway were analyzed, and the enrichment pathways of DEGs were also assessed using a significance threshold of *q* < 0.05.

### 2.6. A Quantitative Reverse-Transcription PCR (qRT-PCR) for the Validation and Analysis of Expression Patterns

Total RNA was extracted from the stems of the different samples (Rm_stock, Rm, Rr_scion, and Rr), as described above. Reverse transcription was performed using Superscript II reverse transcriptase (Invitrogen) according to the manufacturer’s instructions. Reverse transcription was performed using oligo (dT) primers and 1 μg of total RNA. The primer sequences are listed in Supplementary Table S1. To detect transcript abundance, qRT-PCR was performed using an ABI Prism 7500 system (Applied Biosystems, Foster City, CA, USA) and a SYBR Premix Ex Taq kit (TaKaRa, Tokyo, Japan). The qRT-PCR was performed in 20-μL volumes containing 2 μL first-strand cDNA, 200 nM of each primer, and 10 μL of the 2× SYBR PCR mixture, with the following cycling parameters: 95°C for 30 s, 40 cycles of denaturation at 95 °C for 3 s, and annealing and extension at 60°C for 30 s. Three replicates were conducted in parallel, and the results were normalized differentially to the expression level of constitutive *actin* (Rm) or *GAPDH* (Rr). A relative quantitative method (ΔΔCt) was used to evaluate quantitative variation [23].

## 3. Results

### 3.1. Effects of Rosa Grafting on the Stem Growth of Scion and Stock

**Figure 1:**
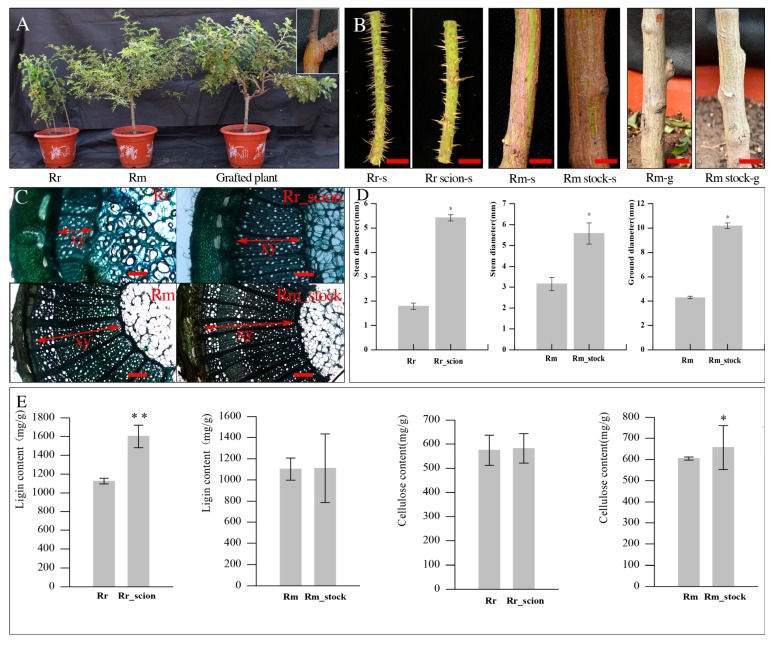
Grafting increases the scion and rootstock stem secondary growth in a *Rosa* grafted plant. (**A**) The seedlings of *R. rugosa* ‘Rosea’ (Rr), *R. multiflora* ‘Innermis’ (Rm), and a grafted plant (Rr grafted on Rm). (**B**) An analysis of stems between *R. rugosa* ‘Rosea’ and *R. rugosa* ‘Rosea’ as the scion, *R. multiflora* ‘Innermis’ and *R. multiflora* ‘Innermis’ grafted, and the root neck between *R. multiflora* ‘Innermis’(Rm) and *R. multiflora* ‘Innermis’ grafted (Rm_stock). Rr (*R. rugosa* ‘Rosea’); Rr_scion (*R. rugosa* ‘Rosea’ grafted); Rm-s (the stem of *R. multiflora* ‘Innermis’); Rm-stock-s (the stem of *R. multiflora* ‘Innermis’ grafted); Rm-g (the root-neck of *R. multiflora* ‘Innermis’); Rm-stock–g (the root-neck of *R. multiflora* ‘Innermis’ grafted). Bars (B): 1 cm (Rr-s, Rr scion-s); 1.5 cm (Rm stock-s and Rm stock–s); 2 cm (Rm-g and Rm stock-g). (**C**) Cross-sections of stems showing the increase in xylem width in Rr_scion vs. Rr and Rm_stock vs. Rm. Scale bars represent 200 µm m in Rr_scion and Rr, 400 µm in Rm_stock and Rm. (**D**) Effect of grafting on the stem thickness between Rr_scion vs. Rr and Rm_stock vs. Rm, and the ground diameter of Rm_stock vs. Rm. (**E**) The lignin and cellulose content in Rr_scion vs. Rr and Rm_stock vs. Rm. Error bars represent ± SD from five independent experiments; * *p* < 0.05, ** *p* < 0.01.

To reveal the effect of grafting on the growth of scion and rootstock and on the stem thickness of Rr_scion (*R*. *rugosa* ‘Rosea’ as scion) and Rm_stock (*R. multiflora* ‘Innermis’ as stock), a phenotype analysis of a cross-section and of the lignin and cellulose content was conducted 1 year after grafting. The stem thickness of Rr_scion (the sixth internode) increased by 47.32% compared with Rr, and the stem (about 5 cm below the grafting point) and stem neck of Rr_scion increased by 291.98% and 206.12%, respectively, compared with Rm (Figure 1). There were no differences in the xylem morphological characteristics in Rr_scion vs. Rr and Rm_stock vs. Rm, but the xylem width in the stem increased by 127.27% and 49.50% in Rr_scion and Rm_stock, respectively (Figure 1). The lignin and cellulose content increased in Rr_scion and Rm_stock; the lignin content was significantly increased, by 42.41%, in Rr_scion vs. Rr, and the cellulose content increased, by 8.77%, in Rm_stock vs. Rm.

### 3.2. Illumina Sequencing and De Novo Assembly of the Grafted Rosa Transcriptome

To investigate the genes associated with grafting response, four cDNA libraries were constructed from total RNA extracted from stems of Rr, Rr_scion, Rm and Rm_stock. The libraries were then sequenced by the Illumina Hiseq4000 platform. Summary of sequence assembly after illumina squencing was shown in Table 1. A tatal of 184,747 transcripts from Rr and 154,572 transcripts from Rm were obtained from clean data. In addition, 136,293 unigenes from Rr and 184,747 unigens from Rm were obtained with an average length of 816 bp and 859bp (Table 2; Table S2). The RNA-seq data can be found in the NCBI Sequence Read Archive (SRA) under ID number PRJNA406996, and the accession of the assembly cDNA data in Rm and Rr are SRR11091586 and SRR11091585.

### 3.3. Analysis of DEGs of R. rugosa ‘Rosea’/R. multiflora ‘Innermis’ Grafts

**Figure 2:**
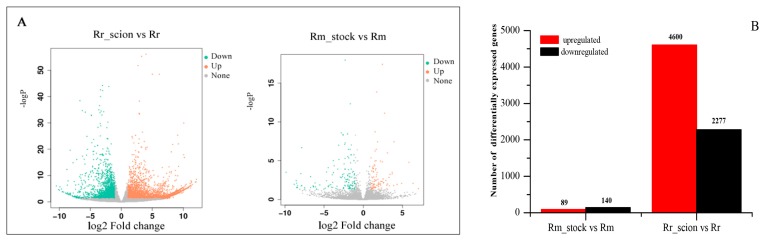
Gene expression profiles of Rm_stock vs. Rm and Rr_scion vs. Rr. (**A**) Volcano map of up- and downregulated genes in Rm_stock vs. Rm and Rr_scion vs. Rr. (**B**) The number of up- and downregulated homologous genes in Rm_stock vs. Rm and Rr_scion vs. Rr. The significance of gene expression differences was determined using *q* ≤ 0.05 and an absolute value of log2 ratio ≥ 1.

Compared with ungrafted samples, there were 6877 and 229 DEGs in Rr_scion and Rm_stock, respectively (*q* < 0.05). In the Rm_stock vs. Rm comparison, 89 DEGs showed increased transcript abundance, and 140 DEGs exhibited decreased transcript abundance (*q* < 0.05). In the Rr_scion vs. Rr comparison, 4600 DEGs were upregulated and 2277 DEGs were downregulated (*q* < 0.05). There were 4511 upregulated DEGs and 2137 decreased transcripts in Rr_scion, more than in Rm_stock, when compared with their control samples. (Figure 2A,B, Table S3). The heatmap of DEGs in Rr_scion vs. Rr and Rm_stock vs. Rm was listed in Figure S1. We also found that 67.25% of DEGS (154 of 229) were successfully annotated, and 22.75% (75 of 229) were unannotated in Rm_stock vs. Rm, whereas 71.62% of DEGs (4925 of 6877) were annotated and 28.38% (1945 of 6877) were unannotated in Rr_scion vs. Rr (Table S4).

### 3.4. GO Enrichment Analysis of DEGs

**Figure 3:**
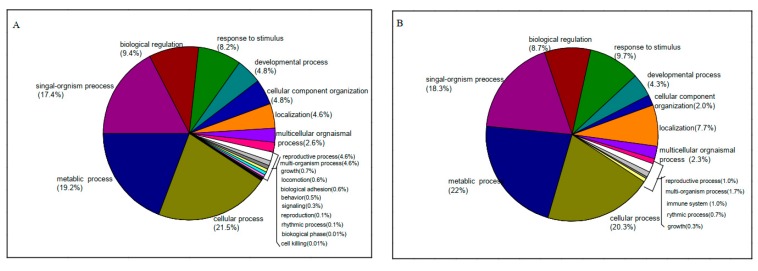
Biological function categories of differentially expressed genes (DEGs) identified in *R. multiflora* ‘Innermis’ after grafting with *R. rugosa* ‘Rosea.’ Gene ontology (GO) categories in comparing Rr_scion vs. Rr (**A**) and Rm_stock vs. Rm (**B**).

After conducting a GO analysis using blast2GO, the contigs of Rr and Rm were classified into 67 terms, and “cellular process,” “cell part,” and “binding” were all dominant among the each of the three main GO classifications “biological process”, ”cellular component” and “molecular function”, (Figure S2). To better understand the function of genes that affected growth and development after grafting, we further analyzed the GO terms of the DEGs enriched between Rr_scion vs. Rr and Rm_stock vs. Rm (Table S5). In the biological process category, these biological processes were mainly associated with “response to stimulus or stress,” “developmental process,” and “multicellular organismal process” between Rr_scion and Rr (*p* < 0.05) (Figure 3; Table S5). We also found that the GO terms “oxidoreductase activity,” “oxygen oxidoreductase activity,” “cellulose synthase,” and “protein serine/threonine kinase activity” in the molecular function category and “plasma membrane” in the cellular component category were enriched in Rr_scion vs. Rr. Among Rm_stock and Rm (control) samples, most of the DEGs enriched in biological process terms were involved in “metabolic process” (GO:0008152, 70 DEGs) and “cellular process” (GO:0009987, 67 DEGs) (Figure 3; Table S5). These cellular components were associated with “intrinsic component of membrane” (GO:0031224, 43 DEGs), and the molecular functions “cytokinin dehydrogenase activity” (GO:0019139, 2DEGs) and “transmembrane transporter activity” (GO:0022857,19DEGs) were observed in Rm_stock vs. Rm. The enriched GO analysis also showed that the scion and rootstock responded differently to grafting (Table S5).

### 3.5. Pathway Analysis of DEGs in the Scion and Rootstock

**Figure 4:**
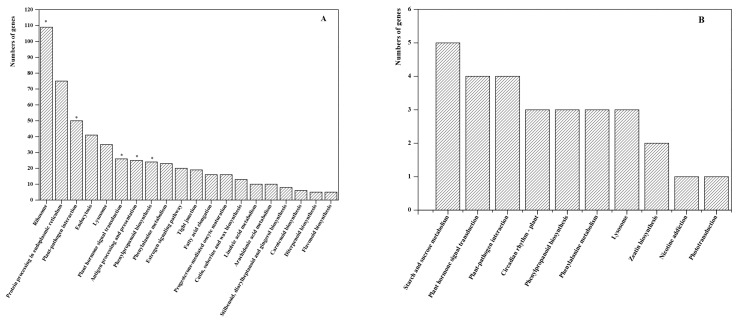
Kyoto Encyclopedia of Genes and Genomes (KEGG) pathway analysis of the differentially expressed genes (DEGs) in Rr_scion vs. Rr and Rm_stock vs. Rm. A total 536 DEGs enriched shown in Rr_scion vs. Rr in order of quantity (**A**); 29 DEGs were enriched in Rm_stock vs. Rm, shown in order of quantity (**B**). * indicates significantly enriched KEGG pathways (*p* < 0.05).

We next analyzed the DEGs enriched in the pathway between Rr_scion vs. Rr and Rm_stock vs. Rm. For the KEGG pathways, 2982 DEGs from Rr_scion vs. Rr and 67 DEGs from Rm_stock vs. Rm were individually annotated. We further found 234 DEGs were significantly enriched in five metabolic pathways in the Rr_scion samples (*p* < 0.05, *q* < 0.05), whereas none of pathways in Rm_stock vs. Rm was enriched significantly (*p* < 0.05, *q* < 0.05). Many KEGGs enriched in Rr_scion vs. Rr “Plant hormone signal transduction” (map04075), “phenylpropanoid biosynthesis” (map00940), and “plant-pathogen interaction” (map04626) were also found in Rm_stock vs. Rm (Figure 4; Table S6); “Starch and sucrose metabolism” (map00500, 5 DEGs) and “Zeatin biosynthesis” (map00908, 2 DEGs) were the specific pathways in which most DEGs were enriched in Rm_stock vs. Rm (Table S6).

### 3.6. Functional Genes in the Scion and Rootstock

#### 3.6.1. Protein Kinases

**Figure 5:**
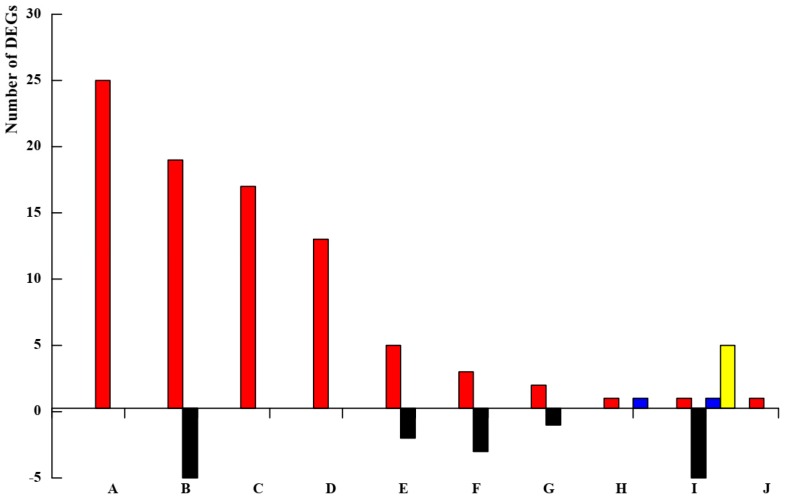
Transcriptional characteristics of differentially expressed genes (DEGs) related to protein kinase. The 10 points (A–J) from left to right on the *x*-axis represent contigs encoding protein kinase in Rm-stock vs. Rm and Rr-scion vs. Rr. Contig encodings: leucine-rich repeat receptor-like serine/threonine protein kinase (A); G-type lectin S-receptor-like serine/threonine protein kinase (B); cysteine-rich receptor-like protein kinase (C); wall-associated receptor kinase (D); L-type lectin domain-containing receptor kinase (E); mitogen-activated protein kinase kinase kinase (F); adenylate kinase (G); receptor protein kinase-like protein (H); leucine-rich repeat receptor-like serine/threonine protein kinase (I); and histidine kinase (J). The red and black colors represent the upregulated and downregulated DEGs encoding different kinds of protein kinase in Rr_scion vs. Rr; The blue and yellow colors represent the upregulated and downregulated DEGs in Rm_stock vs. Rm.

In our study, 103 DEGs were predicted to encode protein kinases according to the functional annotation, of which 96 and 7 contigs encoding protein kinases were differentially expressed in Rr_scion vs. Rr and Rm_stock vs. Rm samples, respectively (Figure 5, Table S7). In Rr_scion vs. Rr, we found that 89.58% of DEGs (86 of 96) encoding protein kinase were upregulated, and 10.42% (10 of 96) were downregulated. We further identified 26 DEGs encoding leucine-rich repeat receptor-like serine/threonine protein kinase (LRR-RLK). Another 32 DEGs encoding cysteine-rich receptor-like protein kinase (CRK), wall-associated receptor kinase (WAK), and receptor-like protein kinase were all differently upregulated; of these, 15 DEGs (including c160972_g4, c177139_g1, c112384_g1, and c112384_g1) were upregulated more than 20-fold (Figure 5, Table S7). For Rm_stock vs. Rm, three categories of DEGs encoding LRR-RLK, receptor protein kinase, and receptor-like cytosolic serine/threonine-protein kinase, were identified, and the four DEGs encoding LRR-RLK were downregulated differently (Figure 5, Table S7). The contrasting expression patterns of LRR-RLK indicated that they were differentially modulated in Rr_scion vs. Rr and Rm_stock vs. Rm.

#### 3.6.2. Transcription Factors (TFs)

**Figure 6:**
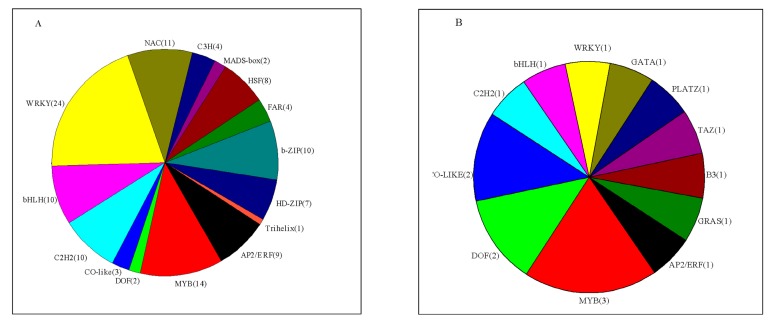
Categories of differentially expressed transcription factors in Rr_scion vs. Rr (**A**) and Rm_stock vs. Rm (**B**). AP2/ERF: ethylene-responsive transcription factor; MYB: MYB transcription factor; DOF: Dof zinc finger protein; Co-like: zinc finger protein CONSTANS-LIKE; C2H2: zinc finger protein; bHLH: transcription factor bHLH36; WRKY: WRKY transcription factor; NAC: NAC transcription factor; C3H: zinc finger CCCH domain-containing protein; MADS-box: B3 domain-containing transcription factor; HSF: heat stress transcription factor; FAR: protein FAR1-RELATED SEQUENCE; b-ZIP: bZIP transcription factor; HD-ZIP: homeobox-leucine zipper protein; Trihelix: trihelix transcription factor; GATA: GRAS domain family; PLAZA: PLATZ transcription factor; TAZ: BTB/POZ domain-containing protein; B3: B3 domain-containing transcription factor; GRAS: GRAS domain family.

A number of TFs have been reported to play important roles in the secondary growth of stems. In our study, 96 differentially expressed transcripts were identified belonging to 15 TF categories in Rr_scion vs. Rr, and 16 transcripts belonging to 12 TF categories were identified in Rm_stock vs. Rm (Figure 6, Table S8). In Rr_scion vs. Rr, the largest number of DEGs was found for WRKY TFs (24 DEGs), followed by zinc finger protein (19 DEGs) and MYB (14 DEGs). We also identified 76 DEGs encoding TFs as being upregulated in Rr_scion vs. Rr, whereas 24 were found to be downregulated. In addition, 94% of DEGs (15 of 16) encoding MYB TFs, DOF TFs, and others were upregulated in Rm_stock vs. Rm (Figure 6, Table S8).

#### 3.6.3. Transporter Genes

Many DEGs encoding different types of transport factors were differentially expressed. In Rm_stock, 19 DEGs were related to the transport of vacuolar amino acids, ABC, sugar, boron, inorganic phosphate, potassium, metals, folate–biopterin, xyloglucan endotransglucosylases/hydrolases, and lipids. The expression of a vacuolar amino acid transporter gene (contig c130686_g1) showed a 15-fold increase in Rm_stock relative to Rm. In Rr_scion, 69 DEGs encoded 18 different categories of transport proteins (Table S9). A total of 16 ABC transporters were significantly differentially expressed in Rr_scion vs. Rr.

### 3.7. qRT-PCR Validation of Differentially Expressed Transcripts from RNA-seq

**Figure 7:**
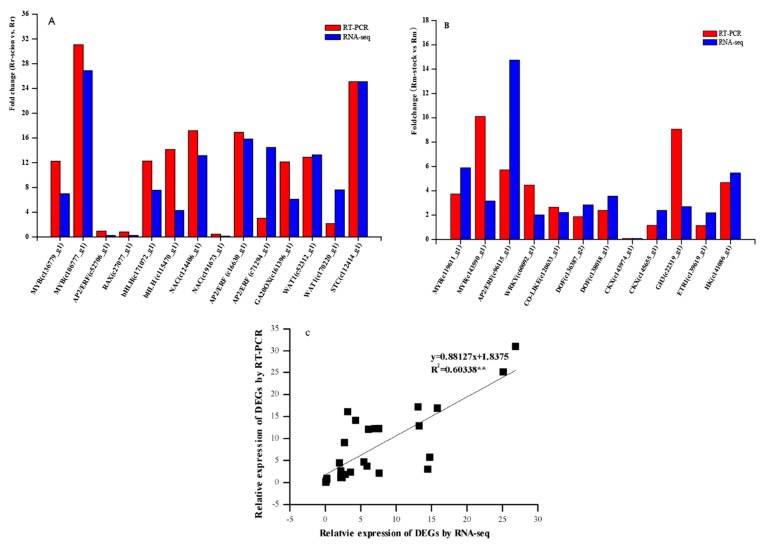
Changes in the transcript levels of 26 selected genes as detected by quantitative reverse-transcription PCR (qRT-PCR). Red bars represent the relative intensity of qRT-PCR from three independent biological replicates, and blue bars represent the expression level of the transcript by RNA-seq. (**A**) 12 qRT-PCR results from Rr_scion vs. Rr.; (**B**) 14 qRT-PCR results from Rm_stock vs. Rm.; (**C**) Coefficient analysis between gene expression ratios obtained from RNA-seq and RT-qPCR data. ** Significant difference at *p* < 0.05. MYB: MYB transcription factor; AP2/ERF: ethylene-responsive transcription factor; RAX: transcription factor RAX3; bHLH: transcription factor bHLH; NAC: NAC transcription factor; GA20OX: gibberellin 20 oxidase; WAT: walls are thin1-related protein; STC: sugar transporter; Co-like: zinc finger protein CONSTANS-LIKE; DOF: Dof zinc finger protein; CKX: cytokinin dehydrogenase; GH3: auxin-responsive GH3; ETR: ethylene receptor.

To confirm the accuracy and reproducibility of our Illumina RNA-seq results, a small number of unigenes, including TFs and hormone-associated genes, were randomly chosen for qRT-PCR amplification. The primer sequences for these 26 unigenes are listed in Table S1. The relative expression levels of these unigenes were analyzed, yielding general agreement in their transcript abundance, as determined by RNA-seq (Figure 7), thus corroborating our RNA-seq transcriptomic data. For example, the contigs c120633_g1 and c141086_g1 in Rm_stock and Rm and the contigs c112414_g1 and c52312_g1 in Rr_scion and Rr showed similar relative differences following the qRT-PCR and RNA-seq. Overall, we found similarity in the expression trends between our qRT-PCR and RNA-seq results (r^2^ = 060), as shown in Figure 7.

## 4. Discussion

### 4.1. Stem Secondary Growth Response for Grafted R. Multiflora ‘Innermis’/R. Rugosa ‘Rosea’ Plants

Rosa is one of the most popular ornamental plants in the world, and the mechanisms of growth, development, and resistance in Rosa are currently the foci of several research projects. The genotypes of the rootstock or scion are often selected for their ability to alter the growth and development of a grafted plant. In our study, the secondary growth (stem thickness) of the scion *R. rugosa* ‘Rosea’ significantly increased compared to an ungrafted control. This result was similar to that of previous studies on grafted apples, grapevines, and watermelons compared with a self-grafted control [11,24,25]. In addition, the stem thickness of the rootstock was significantly increased in the Rm_stock vs. Rm, suggesting that the scion biomass also conferred vigor to the rootstock, similar to the scion genotype’s effect on the shoot and root in grated grape [3].

### 4.2. DEGs in Key Pathway about Lignin and Cellulose Biosynthesis in the Scion and Rootstock

Lignins are complex phenolic polymers of plant cell walls, and reductions in lignin during stem secondary development can cause growth defects and affect water and nutrient transport in plants [26]. Key insights into the molecular aspects of gene regulation in grafted plants can be attained by analyzing DEGs [24]. The phenylpropanoid biosynthesis pathway (map00940), which is related to lignin biosynthesis, was significantly enriched, and DEGs encoding enzymes implicated in lignin synthesis were identified in Rr-scion vs. Rr (Table S10). Peroxidase (PRX), AtPrx2, AtPrx25, and AtPrx71 are involved in lignin biosynthesis, and a significant decrease in the total lignin content has been reported in ATPRX2- and ATPRX25-deficient mutants [27]. These DEGs were confirmed in Rr_scion vs. Rr and Rm_stock vs. Rm (Supplementary Table S10) and were upregulated by 1.34–5.39-fold in both of them. Interestingly, DEGs (60%, 6 DEGs) encoding PRX were downregulated in Rr_scion vs. Rr, which may have regulated the stem lignin structure of the scion [28]. Cinnamyl alcohol dehydrogenase (CAD) catalyzes the last step in the monolignol biosynthesis pathway, and the expression of At4g34230 encoding CAD in Arabidopsis has been reported to increase eight-fold, with very high signal intensity from immature to mature stems [29]. In our study, two DEGs (c158813_g2 and c133940_g1) encoding CAD were upregulated in Rr_scion vs. Rr, however, downregulated in Rm_stock vs. Rm. In addition, we also found that many DEGs encoding 4-coumarate–CoA ligase (CCoAOMT), 4-coumarate–CoA ligase (4-CL) were upregulated in Rr_scion vs. Rr (Table S10). The lignin content increase of Rr_scion also implies that was maybe affected by these genes after grafting (Figure 1) [30].

Cellulose is a major important component of plant cell wall as well as lignin, which are responsible for both oriented cell elongation during plant growth and the strength required to maintain an upright growth habit [31]. Cellulose is a simple polymer of unbranched β-1,4-linked glucan chains [32]. In our study, five DEGs encoding α, α-trehalase, 1,4-α-glucan branching enzyme, and β-amylase were enriched in the “Starch and sucrose mechanism” pathway. It implies the transformation of starch to soluble sugars at the transcriptional level to involved in cellulose synthesis in Rm_stock vs. Rm. In addition, we found DEG (c147623_g4) encoding xyloglucan endotransglucosylase/hydrolase protein (XTH) were upregulated 2.39-fold in Rm_stock vs. Rm, which has been recognized as a cell wall-modifying enzyme. Addition to purified XTH enzyme to Arabidopsis will act predominantly to strengthen or “tighten” cells, or loosen cell walls [33,34]. These DEGs were maybe regulating the stem thickness in Rm_stock vs. Rm, by changing the content of energy production including sugar, cellulose and starch, which need further to be identified [34].

### 4.3. DEGs in Response to Phytohormone Signal Transduction in the Scion and Rootstock

Hormones have been widely applied to regulate secondary vascular growth. Auxin, cytokinins, brassinosteroids, gibberellins, abscisic acid, and ethylene have all been found to control cambial growth and differentiation through a complex regulatory network [35]. In our study, a number of DEGs related to the six hormones mentioned above were identified, and their expression levels were also analyzed in Rr_scion vs. Rr and Rm_stock vs. Rm (Table 3. We found many more DEGs related to hormone signal transduction (e.g., auxin-responsive GH3, EIN3-binding F-box protein EBF, and ethylene receptor ETR) than involved in hormone synthesis pathways (e.g., gibberellin 20 oxidase, cytokinin, and dehydrogenase). This implies that enhanced hormone signal transport and crosstalk may be the main outcome of grafting, rather than increased hormone content. In addition, auxin appeared to play a primary role in vascular patterning in Rr_scion vs. Rr, and the transcriptomes of 18 genes (including AUX/IAA, auxin binding protein, and auxin-induced protein) were significantly changed. They were found to be highly and preferentially expressed in xylem and during the differentiation of tracheary elements [36], and the loss and gain of function resulted in reduced and discontinuous vascular formation, respectively [37,38].

For Rm_stock vs. Rm, the DEGs were more related to cytokinin and indole-3-acetic acid synthesis in the zeatin biosynthesis pathway (map00908). Cytokinin has been recognized as key regulator of cambial activity, procambium maintenance, and development of the vasculature [39]. Degradation of cytokinin is catalyzed by cytokinin oxidase/dehydrogenase (CKX) enzymes. It was identified that ckx3 ckx5 and ckx7 regulated the activity of the reproductive meristems of Arabidopsis [40,41], It was found the inflorescence stem of ckx3 ckx5 mutants is thicker than the wild type, and CKX7-overexpressing in transgenic Arabidopsis reduce the meristem size of roots and induce the cessation of root growth [41]. In our study, two DEGs (c143974_g1 and c145655_g1) were upregulated or downregulated in the stem of Rm_stock vs. Rm, which implied to regulate the development of stem, and the specific regulating should be identified in the future study. In addition, some findings have indicated an important role for long-distance basipetal transport of cytokinin through the phloem in controlling vascular patterning in roots via inhibitory interaction with auxin [42,43], and whether the stem vascular development was affected by cytokinin transportation should be explored. The different expressions of hormone-related DEGs suggested distinct responses in Rr_scion and Rm compared to the control.

### 4.4. Protein Kinases Are Related to Stem Vascular Development in the Scion and Rootstock

Protein kinases (PKs) play an important role in cellular singular transduction processes during plant growth and development [44,45]. In Arabidopsis, 223 LRR-RLK gene expression patterns were identified that had specific characteristics at various developmental stages [46]. We compared the DEGs in Rr_scion vs. Rr and Rm_stock vs. Rm with 14 LRR-RLK subfamilies in Arabidopsis (Figure 7). For Rr_scion vs. Rr, the DEGs (C157420_g1, C152554_g1) were separately assembled into LRRX and LRRIX subfamilies in Arabidopsis, and were upregulated by 3.92 and 12.58-fold (Figure S3A), resulting in increased stem growth and vascular development and increasing the length of petioles and hypocotyl growth due to brassinosteriod activity when they were overexpressed in transgenic Arabidopsis [47,48]. Genes from the LRR-RLKXI subfamily were found to reduce plant growth and affect vascular development when loss of either RLK PHLOEM INTERCALATED WITH XYLEM (pxy) or RLK XYLEM INTERMIXED WITH PHLOEM1 (xip1) occurred. Interestingly, the homologous gene (c144124_g2) was significantly downregulated, by 4.76-fold, in Rm_stock vs. Rm, and was not consistent with the stem thickening growth of the rootstock (Figure S3B). The other DEGs encoding LRR-RLKs (except c9455_g1) were also all downregulated in Rm_stock vs. Rm, suggesting that these genes were negatively related to plant growth and development. In addition, many other protein kinases, such as WAKs, MAPK, LecRKs, GsSRK, and CRKs, were identified as DEGs. Their function in stem secondary growth requires further study.

### 4.5. Transcription Factors Involved in Stem Secondary Growth in the Scion and Rootstock

Furthermore, there is increasing evidence that various TF families, such as MYB, NAC, and WRKY, are involved in the regulation of stem secondary growth [26,49]. Our data provide evidence that some candidate TFs were involved in stem secondary growth after grafting. The NAC and MYB TF genes are the key players regulating the complex transcriptional network leading to wall-thickening cell differentiation [50,51]. We further analyzed NAC and MYB DEGs in our study according to the phylogenetic tree domain in *Arabidopsis*. Two DEGs (c157108_g1 and c130114_g1) were homologous with *AtVND1*, *AtVND7*, and *AtXND1* in Rr_scion vs. Rr (Figure S3C); these play important roles in vessel formation [52,53]. Interestingly, the DEG c109220_g1 encoding MYB TF in Rr_scion vs. Rr and c119611_g1 in Rm_stock vs. Rm were both found to be homologous with *AtMYB75* (Figure S3E,F), which negatively regulated the secondary cell wall (SCW) [54]. It has been suggested that the NAC-MYB-based transcriptional network can regulate the stem secondary growth of Rr_scion and Rm_stock after grafting [55]. In addition, TFs such as the AP2-EREBP, bHLH, C2H2, C2C2-GATA, and GRAS gene families have been found to regulate secondary cell wall metabolic genes through the protein–DNA network in *Arabidopsis thaliana* [56]. In our study, 9 bHLH family, 10 C2H2 family, and 7 HD-ZIP family TFs were identified in Rr_scion vs. Rr, and the C2C2-GATA and GRAS gene families were identified in Rm_stock vs. Rm, providing a number of candidate regulators of stem growth after grafting.

### 4.6. Transporter Proteins Are Important for Stem Secondary Growth in the Scion and Rootstock

Substantial DEGs encoding transporters such as ABC transporter, sugar, boron, inorganic phosphate, and potassium have been found in Rr_scion vs. Rr and Rm_stock vs. Rm. In plants, they are an important membrane transport protein family, relevant to the transportation of hormones, lipids, metal ions, and exogenous substances [57]. Some ABC transporters, especially *AtABCB* (*AtABCB1* and *AtABCB19*) and *AtABCG* (*AtABCG14*, *AtABCG25*, and *AtABCG40*) have been reported to serve a function in stem lignification by auxin, tZ-type cytokinins, and ABA transport and distribution [58,59]. Most DEGs (16 DEGs in Rr_scion; 3 DEGs in Rm_stock) encoding ABC transporter were identified in Rr_scion vs. Rr and Rm_stock vs. Rm. The DEGs (c124372_g1, c150319_g3, and c154902_g1) were homologous with *AtABCG* according to the phylogeny tree (Figure S3H) in Rr_scon vs. Rr; they seemed to function as hormone transporters, affecting the secondary growth of stems after grafting, and should, therefore, be further studied. In addition, sugar transporters (16 DEGs) were more strongly differentially expressed in Rr_scion vs. Rr and Rm_stock vs. Rm. Many sugar transporters localized in phloem companion cells and the associated parenchyma in maturing stems were found to affect vascular development [60,61]. Transporter DEGs may suggest that the growth and development of Rosa grafted plants were altered by the transportation of complex organic materials in Rr_scion and Rm_stock. This has been verified in grafted watermelon plants, where many DEGs are responsible for water transport [24]. Five DEGs were enriched in the KEGG pathway “Starch and sucrose metabolism” (map00500) in Rm_stock vs. Rm, which implies that changes in sugar content may affect the stem growth of rootstock.

Recently, regulatory networks activated in response to different developmental stages and various abiotic and biotic stimuli have been identified [62,63]. Stress responses are likely integrated into the gene regulatory network that determines xylem cell specification and differentiation [56]. Hydrogen peroxide (H_2_O_2_), one of the stress-induced reactive oxygen species (ROS), also plays an important regulatory role in lignin biosynthesis [64]. Ascorbate peroxidase (APX) and CuZn-superoxide dismutase (CuZn-SOD) genes have been found to positively regulate secondary wall biosynthesis and promote growth in Arabidopsis [65]. In addition, the stem lignin metabolism is known to be related to plant disease resistance, insect resistance, and tolerance of drought, salt, heat, cold, heavy metals, and other stresses [66]. In our study, 845 DEGs significantly enriched in 25 GO terms were identified in the biological process “Response to stimulus or stress” in Rr_scion vs. Rr (*p* < 0.05), and 17 DEGs were also involved in Rm_stock vs. Rm. How these stress DEGs are regulated by grafting to improve stem secondary growth requires further investigation. In addition, many related abiotic stimuli DEGs have been identified in other grafted plants such as watermelon and grapevine [11,24].

## 5. Conclusions

Grafting is particularly important for plant development. This study used transcriptome data for the first time to analyze the stem secondary growth of the scion and rootstock after grafting. The scion *R. rugosa* ‘Rosea’ and rootstock *R. multiflora* ‘Innermis’ stem morphology were observed, and the lignin and cellulose content were measured. These were found to be significantly changed compared to an ungrafted control. A total of 136,293 unigenes in Rr and 108,651 unigenes in Rm were obtained, and 6,877 and 229 DEGs were detected, respectively. The data revealed important pathways for stem secondary growth of the scion and rootstock after grafting, such as “Plant hormone signal transduction” and “Phenylpropanoid biosynthesis,” consistent with morphological changes in the scion, and “starch and sucrose metabolism” in the rootstock. Substantial signal transduction genes such as PK, TF, and transporters were found to regulate the secondary cell wall of the stem and may be important determinants of the underlying stem secondary growth. These results will facilitate future analysis of the roles of these genes in stem secondary growth after grafting and will also be useful for enabling rootstock breeding with thicker stems.

## Figures and Tables

**Table 1:** Summary of sequence assembly after illumina squencing for *Rosa multiflora* ‘Innermis’ and *R. rugosa* ‘Rosea’.

Sample	Total Reads	Clean Reads	Clean Base	Error (%)	Q30
Rr	199,710,618	184,953,434	27,743,015,100	0.02	96.82
Rr_scion	198,497,050	184,574,214	27,686,132,100	0.02	96.75
Rm	199,381,448	184,304, 392	27,645,658,800	0.02	96.32
Rm_stock	196,825,416	180,846,594	27,126,989,100	0.02	96.70

**Table 2:** Summary of the transcriptome assembly.

Sample	Transcripts	Unigene	Aligned Reads	GC (%)	Min (nt)	Max (nt)	Mean (nt)	N50	N90
Rr	154,572	136,293	174,417,164	43.80	201	93521	816	1069	286
Rm	184,747	108,651	172,594,386	43.24	201	100409	859	1220	290

**Table 3:** DEG data for selected genes related to regulation of diverse hormones in Rr_scion vs. Rr and Rm_stock vs. Rm.

Rr_scion vs. Rr					
Contig	Grafting	Non-Grafting	Fold Change	Log_2_ Fold Change	Gene Description
c161396_g1	168.94	27.72	6.09	2.619	gibberellin 20 oxidase
c150592_g1	25.44	6.36	3.99	2.00	gibberellin 20 oxidase
c137188_g1	10.864	42.50	0.26534	−1.97	gibberellin 20 oxidase
c139468_g3	29.924	4.65	6.43	2.69	gibberellin 20 oxidase
c169466_g1	1202.19	329	0.36	−1.45	Gasa4-like protein
c145719_g1	29.71	7.28	4.083	2.03	gibberellin 2-β-dioxygenase
c144986_g1	2.30	9.58	0.24	−2.06	auxin transporter-like protein
c80584_g1	1075.22	6844.72	0.16	−2.67	auxin transporter-like protein
c159798_g7	932.27	399.73	2.33	1.22	auxin binding protein
c111040_g1	213.30	43.45	4.91	2.30	auxin binding protein
c170220_g1	315.98	41.63	7.59	2.92	wall are thin1-related protein
c191576_g1	32.29	7.46	4.33	2.11	wall are thin1-related protein
c152340_g1	69.88	30.49	2.29	1.197	wall are thin1-related protein
c81300_g1	747.41	296.19	2.52	1.34	wall are thin1-related protein
c52312_g1	12.708	0.96	13.25	3.73	wall are thin1-related protein
c114045_g1	99.80	40.19	2.48	1.31	auxin-induced protein
c122006_g1	3.03	27.71	0.11	−3.19	auxin-induced protein
c142151_g1	104.22	35.71	2.92	1.55	auxin-induced in root cultures protein
c142151_g2	36.64	13.81	2.65	1.41	auxin-induced in root cultures protein
c162170_g1	24.58	76.66	0.32	−1.64	auxin-induced in root cultures protein
c136754_g1	4.49	0.33	13.757	3.78	indole-3-acetic acid-induced protein
c134374_g1	13.33	30.53	0.44	−1.195	auxin-responsive protein
c21225_g1	69.86	209.07	0.33	−1.58	auxin-responsive protein
c146441_g2	4.44	16.22	0.27	−1.87	auxin efflux carrier component
c140539_g1	105.48	332.63	0.32	−1.66	abscisic acid 8′-hydroxylase
c158586_g1	312.40	56.48	5.53	2.47	abscisic acid 8′-hydroxylase
c122806_g1	35.01	135.65	0.26	−1.95	abscisic acid 8′-hydroxylase
c150342_g1	330.36	24.20	13.65	3.77	abscisic acid receptor
c192915_g1	29.7	7.89	3.77	1.91	abscisic acid receptor
c161435_g1	326.73	52.34	6.24	2.64	abscisic acid receptor
c140884_g1	1942.26	69017	0.28	−1.83	abscisic acid insentive protein
c120991_g2	8.082	25.33	0.32	−1.65	abscisic acid insentive protein
c132075_g1	170.47	83.47	2.04	1.03	cytokinin dehydrogenase
c158300_g2	160.80	59.70	2.69	1.4	two-component response regulator
c152947_g1	215.33	106.11	2.03	1.02	ethylene receptor
c145980_g1	267.47	130.48	2.05	1.04	ethylene receptor
c150029_g1	76.21	17.624	4.32	2.11	reversion-to-ethylene sensitivity
**Rm_stock vs. Rm**					
c143974_g1	9.60	135.88	0.071	−3.82	cytokinin dehydrogenase
c145655_g1	276.45	116.26	2.38	1.25	cytokinin dehydrogenase
c22319_g1	27.75	10.31	2.69	1.43	auxin-responsive GH3 gene family
c139619_g1	953.89	435.35	2.19	1.13	ethylene receptor
C140747_g1	375.42	147.53	2.54	1.35	EIN3-binding F-box protein

## Supplementary Materials

The following are available online at https://www.mdpi.com/2073-4425/11/2/228/s1, Figure S1: GO annotation of unigenes in *R. rugosa* ‘Rosea’ (A) and *R. multiflora* ‘Innermis’ (B). Figure S2: COG function classification of *R. rugosa* ‘Rosea’ (A) and *R. multiflora* ‘Innermis’ (B). Figure S3: The homologous genes in Arabidopsis thaliana of the categories of DEGs (LRR, NAC, MYB, ABC transporter) between Rr_scion vs. Rr and Rm_stock vs. Rm. Table S1: the List of RT-PCR primer of *Rosa multiflora* ‘Innermis’ and *R.rugosa* ‘rease’. Table S2: The list of de-novo data and assembled results of *Rosa multiflora* ‘Inermis’ (Rm) and *R.rugosa* ‘Resea’.(Rr). Table S3: The list of the numbers of functional annotation of *R.multiflora* ‘Innermis’_and *R.rugosa* ‘Rease’. Table S4: Functional annotation of DEGs and unknown DEGs in Rm_stock vs. Rm and Rr_scion vs. Rr. Table S5: the list of GO categories of DEGs in GO in Rm-stock vs. Rm and Rr-scion vs. Rr. Table S6: KEGG pathways significanlty enriched in Rm-stock vs. Rm and Rr-scion vs. Rr. Table S7: Differently expressed unigenes related to Protein kinase in Rm_stock vs. Rm and Rr_scion vs. Rr. Table S8: Differently expressed unigenes related to Transcription factor in Rm-stock vs. Rm and Rr-scion vs. Rr. Table S9: Differently expressed unigenes related to transporter genes in Rm_stock vs. Rm and Rr_scion vs. Rr. Table S10: The list of unigenes invovled in Phenylpropanoid biosynthesis in Rr_scion vs. Rr and Rm_stock vs. Rm.
