# Supplementary material for: Comparative Transcriptome Analysis Reveals Stem Secondary Growth of Grafted Rosa rugosa ‘Rosea’ Scion and R. multiflora ‘Innermis’ Rootstock
Source: Genes (Basel). 2020 Feb 21;11(2):228. doi: 10.3390/genes11020228 (PMC7073730; doi:10.3390/genes11020228)
Supplement: Supplementary file 1 [file genes-11-00228-s001.zip › SUPPLEMENTARY/Fig S1-3.docx]

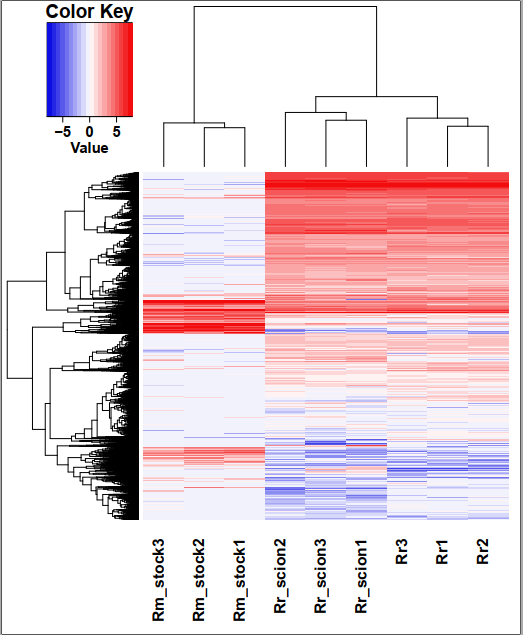

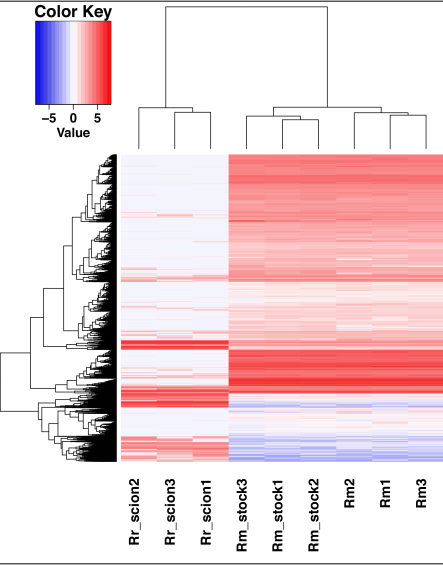


B

A

Fig.S1 Heatmap of DEGs in Rr_scion vs. Rr (A) and Rm_stock vs. Rm (B). The significance of gene expression differences was determined using *q* ≤ 0.05 and an absolute value of log2 ratio ≥ 1.

**Fig. S2** GO annotation of unigenes in *R. rugosa* ‘Rosea’ (A) and *R. multiflora* ‘Innermis’ (B)

A

B

C

D

E

F

G

H

Fig S3. The homologous genes in *Arabidopsis thaliana* of the categories of DEGs (LRR, NAC, MYB, ABC transporter) between Rr_scion vs Rr and Rm_stock vs Rm.(A: LRR, Rr_scion vs Rr; B: LRR, Rm_stock vs Rm; C: NAC, Rr_scion vs Rr; D: NAC, Rm_stock vs Rm; E; MYB, Rr_scion vs Rr; F: MYB, Rm_stock vs Rm; G: ABC transpoter, Rr_scion vs Rr; H: ABC transpoter, Rm_stock vs Rm;).The phylogenetic analysis were performed by using Molecular Evolutionary Genetics Analysis software package, version 7.0 (MEGA 7.0) with the Stastical method of Neighbor-joining (NJ). Bootstrap analysis with 500 replications was performed to assess group support.
